# Genome-wide association studies in psychiatry: Current perspectives

**DOI:** 10.1192/j.eurpsy.2021.219

**Published:** 2021-08-13

**Authors:** D. Smajlagic, T. Zayats, M. Bekkehus, S. Le Hellard

**Affiliations:** 1 Clinical Sciences, University of Bergen, Bergen, Norway; 2 Analytic And Translational Genetics Unit, Massachussets General Hospital, Bostton, United States of America; 3 Department Of Psychology, University of Oslo, Oslo, Norway

## Abstract

**Abstract Body:**

Genome-wide Association Studies in Psychiatry - Current Perspectives Last decade was exciting time for human genetic studies. Genome-wide association studies (GWASs), used to examine the association of genotyped variants across the entire genome and common complex phenotype(s), have led to many findings. Currently, GWAS Catalogue has collected 4,809 publications and 227,262 associated variants. In recent years, psychiatric genetics has enjoyed some success in discoveries of associated variants. This mostly happened because researchers were able to unite and generate large sample sets of patients and healthy controls in big consortia. As a result of large sample sizes becoming available for meta-GWASs, some of the first genome-wide significant loci in psychiatric and related neurodevelopmental traits were detected. However, most of the large-scale genetic studies are done primarily on European population and GWASs have huge diversity problem. Performing trans-ethnic GWASs on psychiatric traits can help us discover more associated variants. Another advantage of bringing many datasets together into large-scale meta-analyses is the ability to conduct cross-disorder studies. This is possible to be done on psychiatric traits since many of them share genetic liability. However, little research has been conducted on the genetic differences between related psychiatric traits. Identifying disorder-specific variants remain important open question. In this presentation we will bring an update of recent findings and current state of the art methods and analyses.

**Disclosure:**

No significant relationships.

